# Establishment of a Pharmacogenetics Service Focused on Optimizing Existing Pharmacogenetic Testing at a Large Academic Health Center

**DOI:** 10.3390/jpm10040154

**Published:** 2020-10-03

**Authors:** Amy L. Pasternak, Kristen M. Ward, Mohammad B. Ateya, Hae Mi Choe, Amy N. Thompson, John S. Clark, Vicki Ellingrod

**Affiliations:** Department of Clinical Pharmacy, College of Pharmacy, University of Michigan, Ann Arbor, MI 48109, USA; kmwiese@med.umich.edu (K.M.W.); Mohammad.Ateya@pfizer.com (M.B.A.); haemi@med.umich.edu (H.M.C.); amynt@med.umich.edu (A.N.T.); johnclar@med.umich.edu (J.S.C.); vellingr@med.umich.edu (V.E.)

**Keywords:** pharmacogenetics, pharmacogenomics, implementation, pharmacogenetics service

## Abstract

Multiple groups have described strategies for clinical implementation of pharmacogenetics (PGx) that often include internal laboratory tests that are specifically developed for their implementation needs. However, many institutions are not able to follow this practice and instead must utilize external laboratories to obtain PGx testing results. As each external laboratory might have different ordering and reporting workflows, consistent reporting and storing of PGx results within the medical record can be a challenge. This might result in patient safety concerns as important PGx information might not be easily identifiable at the point of current or future prescribing. Herein, we describe initial PGx clinical implementation efforts at a large academic medical center, focusing on optimizing three different test ordering workflows and two distinct result reporting strategies. From this, we identified common issues such as variable reporting location and structure of PGx results, as well as duplicate PGx testing. We identified several opportunities to optimize our current processes, including—(1) PGx laboratory stewardship, (2) increasing visibility of PGx tests, and (3) clinician and patient education. Key to the success was the importance of engaging clinician, informatics, and pathology stakeholders, as we developed interventions to improve our PGX implementation processes.

## 1. Introduction

Pharmacogenetics (PGx) is a pillar of precision medicine that aims to improve healthcare by using genetics to guide prescribing towards safer, more effective medication outcomes. Examples of PGx include testing for genetic variants in human leukocyte antigen (HLA) presenting genes, to decrease the risk of serious adverse drug reactions associated with drugs like abacavir and carbamazepine, and testing for genetic variability in drug metabolizing enzymes, to guide antidepressant dosing. Despite clinical guidelines and Food and Drug Administration-approved package labeling that provides recommendations for select medications based on genotype, pharmacogenetic implementation efforts across the United States are varied in depth and scope [1,2]. Pharmacogenetic implementation pioneers frequently developed research-based programs where patients consent to broad panel-based testing that is integrated into electronic systems, to guide drug prescribing [3,4,5,6]. Others developed inpatient clinical programs where single drug-gene pairs were selected and implemented within a specific practice setting, such as CYP2C19 testing for percutaneous coronary intervention [7,8,9]. Some organizations also developed ambulatory pharmacogenetics services where patients are referred to pharmacogenetics clinics to help guide and interpret pharmacogenetic testing [10,11]. In the majority of cases of these early adopters, the institutions identified a single source for PGx testing, such as internal PGx testing panels, which were customized to the needs of their project, institution, and population.

However, it is unlikely that all health systems will have the capability to establish an internal pharmacogenetics laboratory, and therefore the majority of clinicians will likely utilize commercial laboratories, where pharmacogenetic test offerings are increasing [12]. Without dedicated pharmacogenetics oversight, each clinical specialty within the institution might select a different laboratory and develop their own test, ordering and reporting the workflow for pharmacogenetic results in the electronic medical record (EMR). Multiple independent processes confound the integration of pharmacogenetics into prescribing decisions, and these independent processes might cause decreased visibility of relevant test results to all clinicians providing care to the patient, especially when the results are returned as unstructured text documents, such as Portable Document Format (PDF) files. Multiple groups are creating resources to enable uniform discrete reporting of genetic results in the EMR, however, widespread adoption is not yet achieved [13,14,15]. Consistent result visibility is critical to ensuring appropriate and safe medication use.

Our institution began a pharmacogenetics service in 2018, with the hiring of two clinical pharmacist specialists focused in PGx implementation. However, prior to the initiation of this service several clinical service lines already utilized pharmacogenetic testing. We chose to use an evidence-based approach to determine initial PGx interventions. Therefore, our service focused on developing standardized strategies for incorporating existing PGx results into the EMR, for all relevant patient care decisions for gene-drug pairs, with established recommendations for dosing or use, based on Clinical Pharmacogenetics Implementation Consortium (CPIC) guidelines or Food and Drug Administration (FDA) package insert information. The description of these implementation efforts are unique because the strategies for evaluating and integrating existing PGx results are less well described than implementation of a new PGx-service that sets the testing criteria [16]. We believe that describing our PGx implementation strategy will be informative for clinicians at institutions that are encountering external PGx testing. Our processes might help guide opportunities for PGx result integration when their institution might not have the infrastructure to develop their own pharmacogenetic testing platforms or large-scale informatics efforts. Herein, we describe our initial processes to identify and execute PGx-focused interventions, through the optimization of existing pharmacogenetic testing strategies, implemented at a large academic medical center.

## 2. Materials and Methods

A retrospective review of pharmacogenetics utilization across the health system was designed to assist the team with identifying areas where interventions could be made to optimize or expand existing workflows, improve patient safety, or identify areas for increased education to clinicians. Our goals were to—(1) identify what PGx tests were being ordered within the institution, (2) determine the ordering and return the location of the PGx results, and (3) identify what clinical specialties utilized PGx tests.

After obtaining approval from the institution’s internal review board (HUM00143486), the EMR was queried for PGx test results from 1 June 2014–31 December 2018. Discrete variables were identified through DataDirect, an internal, electronic data repository that extracts discrete information from the institutional EMR [17]. The Electronic Medical Record Search Engine (EMERSE), a free-text search engine of the EMR, was used to extract additional data of interest that could not be captured as a discrete variable (e.g., clinical note documentation or text reported lab results) [18]. In both systems, preliminary searches of laboratory tests, problem list entries, and clinical notes began by using names of germline pharmacogenes, with guidelines from CPIC or germline genes included in the FDA Pharmacogenomic Biomarkers table. Searches were then expanded to include names of pharmacogenetic testing panels, such as Genesight^®^, to further identify cases of commercial pharmacogenetic tests, ordered as a panel.

Once a PGx result was identified in the medical record, additional data were gathered for each result, such as, ordering workflow, testing laboratory (internal vs. external), location of result storage in EMR, and format of result in EMR (i.e., discrete vs. text).

In addition to the retrospective chart review, the pharmacogenetics team began informally surveying providers about their use and perceptions of pharmacogenetics, to determine what services would be beneficial to clinicians and identify opportunities for education. A pharmacogenetic consult service was also established and advertised to providers at our institution. This service is available for any questions related to pharmacogenetics from providers and their patients. Direct consults with patients can be requested by the provider for either pre- or post-PGx testing and are complete via telephone. Clinicians can also request post-result interpretations of pharmacogenetics tests ordered for patients. These interpretations are completed by a clinical pharmacy specialist and returned via a standard pharmacogenetic note template that includes genotype and CPIC phenotype interpretation, in addition to patient-specific considerations for prescribing.

## 3. Results

### 3.1. Retrospective Chart Review

Between 1 June 2014 and 31 December 2018, 6302 pharmacogenetic test results were identified for 5663 patients. Thirteen unique pharmacogenes and 16 unique pharmacogenetic test orders were identified in the EMR (Table 1). Thiopurine methyltransferase (*TPMT*) was the most commonly tested pharmacogene, accounting for 50.6% of all PGx tests ordered. *TPMT* also had the most test order options, with three distinct tests orderable in the EMR, two enzyme activity assays and one genotype test.

Three unique test ordering processes were identified—(1) discrete order in EMR, meaning the test order could be searched in the EMR, (2) non-discrete order in EMR, meaning the test result was within the EMR but the test was placed as a “miscellaneous” order, and (3) external-to-EMR orders, where the test order was placed directly through the commercial laboratory. Ten of the 16 PGx tests were available as discrete orders in the EMR, 5 were available as non-discrete orders in the EMR, and one was ordered external to the EMR.

Of the 15 PGx tests that could be ordered in the EMR, four were performed by an internal laboratory and all were discrete orders. The remaining EMR-orderable PGx tests, whether discrete or non-discrete, were sent to external laboratories. All tests that were ordered within the EMR had the test results displayed within the results section of the EMR. Two of the PGx test results were reported as a discrete genotype or phenotype (*IL28B* and G6PD), while all other PGx test results were reported in the laboratory results, as text comments based on the original laboratory report.

The only way to identify that an external-to-EMR PGx test was completed for a patient was to search for clinician documentation in an encounter note. All identified cases were for a pharmacogenetic panel test that was focused on psychotropic prescribing, and searching for the name of the test panel was the most efficient way to identify cases. In 58% of the identified cases, the genotype result for the patient was identifiable, most commonly through a scanned PDF of the laboratory report that was uploaded into the EMR. Although clinicians mentioned testing was performed in clinical notes, they rarely reported the genotype results for the patient in the associated documentation.

The overall volume of PGx tests did not vary from year to year, although the proportion of *IL28B* tests decreased over the study period, while the proportion of Genesight^®^ panel tests increased over the study period, likely reflecting practice changes over the study period. 

#### 3.1.1. Duplicate Test Results

Although the median number of pharmacogenetic tests per subject was 1, the range of tests per subject was 1–8. Therefore, we evaluated the prevalence of duplicate pharmacogenetic tests.

Overall, 12% of patients (*n* = 680) had >1 result for the same pharmacogenetic test in the EMR, during our study time frame. This was most common for patients with TPMT testing (15%), followed by those with G6PD testing (6.8%), then HLA-B*57:01 (3%), and *UGT1A1* genotype testing (2.2%). The median number of duplicate tests per patient was 2 (range 2–8). For HLA-B*57:01, and *UGT1A1* genotypes, 100% of the duplicate orders occurred during unique patient appointments. For TPMT, 73% of duplicate test orders occurred in unique appointments, while the remainder of duplicate tests were ordered at the same appointment. Sixty percent (*n* = 319) of patients with > 1 TPMT test had multiple TPMT enzyme assay tests, 33% had an enzyme assay and genotype test, and the remainder had multiple TPMT enzyme assays and a genotype test. The large proportion of testing repeated at separate patient appointments suggests the first test result might have been missed by the ordering clinician.

#### 3.1.2. Pharmacogenetic Problem List Entries

Seventy-seven subjects had pharmacogenetic problem list entries for 13 different pharmacogenes (Table 2). A corresponding pharmacogenetic test result was identified in the EMR for 54 (70%) of these PGx problem list entries. The majority of the problem list entries provided information about the gene that was tested, but limited the information about the identified genetic variant or phenotype to allow for clinical application. 

### 3.2. Clinical Services

**Figure 1:**
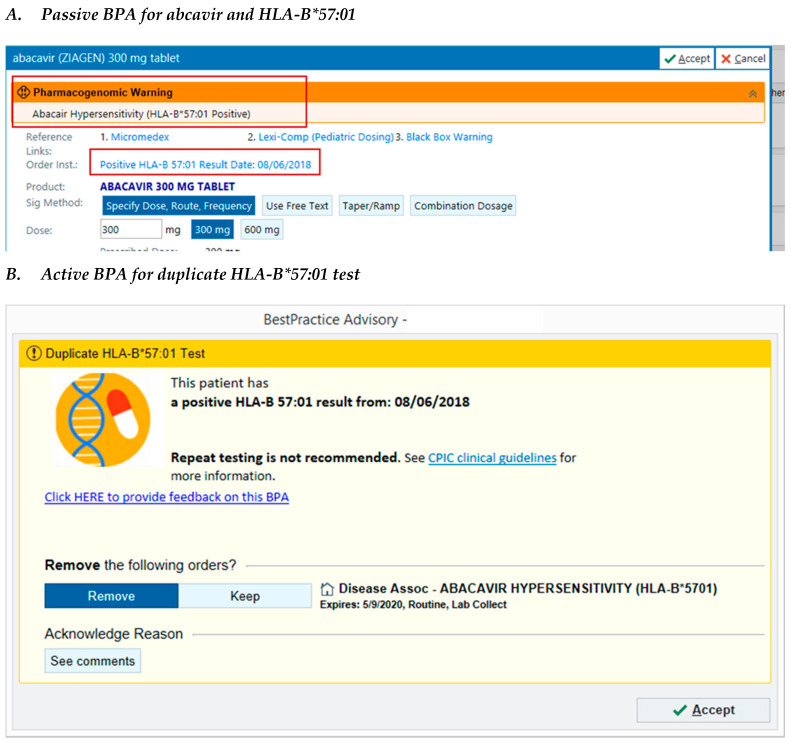
Sample screenshots of pharmacogenetic best practice advisory (BPA) alerts. © 2020 Epic Systems Corporation.

Based on the findings of the retrospective evaluation, we developed additional pharmacogenetic services in the form of clinical decision support to improve PGx-associated workflows. To improve result visibility, we added the relevant pharmacogenetic test result to the medication order screens for abacavir and thiopurines. To address the high rates of duplicate testing, we began the development of clinical decision support, which was implemented for HLA-B*57:01 and *TPMT*. Both passive and active clinical decision support (CDS) strategies were used to notify clinicians that a pharmacogenetic test result was either missing for a relevant medication order, or was already available for a duplicate laboratory order (Figure 1). CDS was also developed to notify clinicians of a high-risk result for the HLA-B*57:01 genotype, which was reported as an unstructured text comment in the EMR. Using custom structured query language, test results were extracted and were subsequently stored in the EMR as discrete data elements. We compared the rate of patients with a duplicate TPMT test order for 6 months pre- and post-CDS implementation. In the pre-CDS period (1 April 2019–1 October 2019), for 17.6% of patients, the TPMT test order placed in this time period was a duplicate test; in the post-CDS period only 9.6% of patients had a duplicate TPMT order placed. No duplicate test alerts fired for HLA-B*57:01 in the post-CDS time period.

While evaluating the discrete test orders, we also identified there was a cost difference between the two available TPMT enzyme assays, with no strong clinical indication to prefer one test from the other. We therefore worked with the laboratory formulary committee and clinicians who utilized this testing to decrease the number of TPMT enzyme assay orders in the EMR, to decrease the overall costs of testing. The overall estimated cost savings for the institution based on these interventions was approximately $47,000 annually.

In addition to clinical decision support, the pharmacogenetics service provided both education and clinical consultation, based on the findings of our initial inquiries. In terms of educational efforts, a grand rounds presentation was provided to the department of pharmacy, as well as small group education with pharmacists on the CDS interventions discussed above. Education and outreach efforts with non-pharmacists were primarily focused on services that utilized the external-to-EMR test orders. The education sessions varied, but frequently covered a review of pharmacogenetics, introduction to pharmacogenetics resources such as the Clinical Pharmacogenetics Implementation Consortium, discussion on how to interpret PGx test results, and potential limitations of pharmacogenetics. Approximately 200 clinical pharmacogenetic consults were completed to date via the consult service, primarily in ambulatory psychiatry, for assistance with the interpretation of commercial laboratory psychotropic pharmacogenetic panels.

## 4. Discussion

Through this investigation, our team identified multiple opportunities for pharmacogenetic interventions to optimize pharmacogenetic testing strategies that already existed within our institution, and to increase the integration of these results into prescribing decisions. There was substantial heterogeneity in terms of both the test ordering and test resulting procedures within our institution. Additionally, we unexpectedly discovered that many patients had duplicate pharmacogenetic testing performed. These findings were not previously described in the pharmacogenetics implementation literature, but are likely true at many institutions where the clinical service lines developed independent strategies for using PGx testing. All of these discoveries present opportunities for pharmacist-led, PGx-focused interventions that have the potential to decrease costs and improve patient safety. 

Laboratory stewardship is the process of improving patient safety by ensuring that appropriate tests are ordered, returned, and interpreted correctly for patients, while maintaining and developing testing protocols that are fiscally responsible [19]. Pharmacists, and other PGx-trained clinicians, can play a significant role in PGx laboratory stewardship within their institutions, by helping to identify inappropriate testing, as well as comparing different testing strategies. This could present opportunities to improve test ordering and resulting workflows, as well as identify cost-saving opportunities, such as our intervention to remove a more expensive, but clinically comparable, TPMT enzyme assay. Although this process does not directly impact the daily pharmacist workflows, it helped to develop and establish mutually beneficial projects for pharmacy, pathology, and clinicians. 

Ideally, all PGx results would be available in a discrete format in one location in the EMR, however, there are substantial barriers to deploying this strategy that might not make it feasible at all institutions. Use of pharmacogenetics is likely to increase and so pharmacists should work to develop strategies to document pharmacogenetic results into the EMR, regardless of the testing source, to improve result visibility and ease communication of test implications. Our initial strategy for improving documentation is a standardized note template that includes the genotype result and uses standardized CPIC phenotype terminology. One primary goal of the result interpretation was to specifically address issues related to psychotropic panel testing. First, the products currently used by our providers only describe pharmacogenetic guided recommendations for psychotropic medications, when the PGx result might be applicable to other drug classes. An example is *CYP2C19* testing, where clinical recommendations currently exist for psychotropic, cardiovascular, and antifungal agents [20,21,22,23]. Providers might not be aware of the non-psychotropic implications of the PGx result and these potentially significant drug–gene interactions might be missed. Secondly, the laboratory interpretations do not consider other patient-specific factors that might impact result interpretation of pharmacogenetics, such as renal and hepatic function and drug–drug interactions. Finally, many of the genetic results are not consistently interpreted into pharmacogenetic phenotypes by different labs [24]. This results in variable interpretations that are sometimes at odds with recommendations from pharmacogenetic guidelines. As our consultation translates the genotype result into a phenotype, based on the CPIC standardized phenotype definitions, results for all patients with consultations show a consistent interpretation. Although this process has limitations, it overcomes many barriers to the traditional storage of PDF lab reports, in that, it is searchable in the EMR, improves the visibility of the genetic results, and overcomes the barrier of variable phenotype interpretations by commercial laboratories that could be inconsistent with interpretations from pharmacogenetic guideline organizations [24].

As described by others, when implementing new clinical services, each of these interventions required the engagement and buy-in of relevant stakeholders. The first step of clinician engagement was educating them on the current state of testing in their practices and presenting potential interventions to optimize the existing process. Once clinician buy-in was achieved, we then engaged pathology and health informatics to further evaluate and approve these interventions. Many PGx programs described establishing pharmacogenetics oversight committees that include stakeholders and provide approvals for all PGx-related testing and interventions [3,25]. The development of this type of oversight committee might represent a barrier to PGx implementation for some institutions, as it might not fit in the existing committee structure or might have too much overlap with other existing committees. We demonstrated that pharmacogenetic interventions can be successfully deployed without establishing a PGx-focused oversight committee, as long as all relevant stakeholders are involved in the development of the interventions. 

There are some limitations to the approach we took to conduct our retrospective review of pharmacogenetics at our institution. Although the queries were completed with extensive terminology in multiple data tracking systems, cases of PGx testing might still have been missed. The EMERSE system helps to minimize this risk, by allowing for “synonym” searches for common alternative or misspellings of the query word, however, some terms might still have been missed in the clinician documentation [18]. Additionally, external results are frequently stored as scanned PDFs in the EMR and there is currently no query method to evaluate this PDF data at our institution. This complication implies that it is likely that additional cases of both single-gene and panel-pgx tests could have been missed in this preliminary search, if they were not also reported in the clinical note format. As this is a challenge many institutions likely face, it highlights how clinicians need to be proactive in identifying what PGx testing is occurring within their practice areas and across their institution to ensure they can be incorporated into relevant patient-care decisions.

Until pharmacogenetic tests are reported as discrete results from all laboratories into all EMRs, interim strategies for capturing pharmacogenetic results will be needed. Clinicians have, and likely will continue, to independently integrate relevant PGx tests into their practices as new PGx associations are discovered. Pharmacists and other PGx-focused clinicians can have a significant impact in optimizing the use of pharmacogenetic tests within their institutions, by contributing to laboratory stewardship, providing education, and providing support for patients and providers on PGx result interpretation.

## 5. Conclusions

Herein, we described the initial processes we developed to establish a PGx-service focused on optimizing the workflows and visibility of existing PGx test orders within our institution. We were able to establish a consult service, with a standard documentation strategy to improve result visibility and develop CDS tools within the EMR, to identify patients who might require PGx testing and prevent duplicate PGx test orders. Successful implementation of services required an assessment of PGx utilization, engagement, and support of relevant stakeholders, and collaboration with informatics. Ideally further integration of test results into the EMR as discrete data would allow for additional CDS development, particularly for results from external laboratories.

## Figures and Tables

**Table 1:** Pharmacogenetic tests identified in electronic medical record from 1 June 2014–31 December 2018.

Test	N (%)	Laboratory	Order Process	Result Location	Result Format
TPMT enzyme assay	2694 (42.7)	External	Discrete	EMR Results	Text
G6PD activity	2122 (33.7)	Internal	Discrete	EMR Results	Discrete
HLA-B*57:01	579 (9.2)	Internal	Discrete	EMR Results	Text
*TPMT* Genotype	496 (7.9)	External	Discrete	EMR Results	Text
Genesight^®^	200 (3.2)	External	External	Clinical Note/Media	NA
*UGT1A1* Genotype	178 (2.8)	Internal	Discrete	EMR Results	Text
*IL28B* Genotype	15 (0.2)	External	Discrete	EMR Results	Discrete
HLA-B*15:02	5 (0.08)	Internal	Discrete	EMR Results	Text
*DPYD* Genotype	5 (0.08)	External	Non-discrete	EMR Results	Text
*CYP2D6* Genotype	4 (0.06)	External	Non-discrete	EMR Results	Text
*CYP2C9/VKORC1* genotype	2 (0.03)	External	Non-discrete	EMR Results	Text
HLA-B*58:01	1 (0.02)	External	Non-discrete	EMR Results	Text
Drug metabolizing enzyme panel	1 (0.02)	External	Non-discrete	EMR Results	Text

EMR—electronic medical record, discrete—reportable and measurable data in EMR, and non-discrete—non-measurable data in EMR.

**Table 2:** Pharmacogenetic problem listing the entries identified in electronic medical record (EMR).

Gene	Problem List Entry	N
*TPMT*	Intermediate TPMT activity	33
	TPMT intermediate metabolizer	1
	Poor metabolizer of azathioprine	1
	Thiopurine methytransferase deficiency	1
*RYR1*	Monoallelic mutation of *RYR1*	14
	Biallelic mutation of *RYR1*	2
*CYP2D6*	CYP2D6 deficiency	2
	Cytochrome p450 2D6 enzyme deficiency	2
	Poor drug metabolizer due to cytochrome p450 CYP2D6 variant	2
*DPD*	DPD Deficiency	6
*CYP2C9*	Monoallelic mutation of *CYP2C9* gene	1
	CYP2C9 deficiency	2
*CYP3A4*	Ultra-rapid metabolizer associated with CYP3A4	2
	Cytochrome p450 3A4 enzyme deficiency	1
*CACN1S*	Monoallelic mutation in *CACN1S*	2
*CYP1A2*	*CYP1A2* gene mutation	2
*CYP2C19*	CYP2C19 intermediate metabolizer	1
	Cytochrome p450 2C19 enzyme deficiency	1
CYP mutation	CYP gene mutation – unknown type	1
	Mutation of liver cytochrome that can lead to impaired drug metabolism	1
*MTHFR*	Biallelic mutation of *MTHFR* gene	1
*CYP2B6*	CYP2B6 intermediate metabolizer	1
*CYP3A5*	*CYP3A5* gene mutation	1
